# 

**Published:** 1898-08

**Authors:** 


					﻿I—
cd
s
go
I—
CD
>-
CD
h—
b~
GO
LU
OE
LU
I—
CD
LU
Q_
C/9
Lu
C9
LU
QC
GO
LU
CD
*c
O_
cs
^9
CE
LU
>-
■«c
CD
I
e
h-I
A
W
>-<
K
iJ
A
i—i
PS
©
QO
oe
b—I
H
I
m
c.
O
C
D
c,
Z
C
>
<
co
(0
(0
-n
O
jj
£
•
o
o
■
SERIES OF STATE EDITIONS.
LEADS VETERINARY JOURNALISM IN AMERICA.
Vol. XIX.
AUGUST, 1898.

PUBLISHED MONTHLY AT $3 A YEAR. SINGLE COPIES, 30 CENTS.
FOREIGN SUBSCRIPTIONS $3.50 PER YEAR.	•'
THE JOURNAL
OF
Comparative Medicine
AND
VETERINARYARCHIVES
EDITED AND PUBLISHED BY ______________
RUSH SHIPPEN HUIDEKOPER, Veteriiratriah fcAlftjS),
. W. HORACE HOSKINS, D.V.SJ-*- D _*
H. D. GILL, v.s.SURG£ON GENtR
ALL COMMUNICATIONS AND PAYMENTS SHOULD BE ADDRESSED TO
Dr. W. Horace Hoskins, Managing Editor, 3452 Ludlow St., Philadelphia, Pa.
Copies may be had on order of all news-dealers at home and abroad.
AN ADVERTISING MEDIUM UNEQUALLED IN THE VETERINARY FIELD.
				

